# Development of a KRAS-Associated Metabolic Risk Model for Prognostic Prediction in Pancreatic Cancer

**DOI:** 10.1155/2021/9949272

**Published:** 2021-10-07

**Authors:** Zuyi Ma, Zhenchong Li, Zuguang Ma, Zixuan Zhou, Hongkai Zhuang, Chunsheng Liu, Bowen Huang, Yiping Zou, Zehao Zheng, LinLing Yang, Yuanfeng Gong, Shanzhou Huang, Qi Zhou, Chuanzhao Zhang, Baohua Hou

**Affiliations:** ^1^Shantou University of Medical College, Shantou 515000, China; ^2^Department of General Surgery, Guangdong Provincial People's Hospital, Guangdong Academy of Medical Sciences, Guangzhou 510080, China; ^3^South China University of Technology School of Medicine, Guangzhou 51000, China; ^4^Sanshui Disease Prevention Cure Station, Foshan 528100, China; ^5^Department of General Surgery, Peking Union Medical College Hospital, Chinese Academy of Medical Sciences and Peking Union Medical College, Beijing 100730, China; ^6^Guangzhou Medical University, Guangzhou 511436, China; ^7^Department of General Surgery, Hui Ya Hospital of The First Affiliated Hospital, Sun Yat-sen University, Huizhou 516081, China; ^8^Department of Liver Surgery, The First Affiliated Hospital of Sun Yat-sen University, Guangzhou 510000, China

## Abstract

**Background:**

KRAS was reported to affect some metabolic genes and promote metabolic reprogramming in solid tumors. However, there was no comprehensive analysis to explore KRAS-associated metabolic signature or risk model for pancreatic cancer (PC).

**Methods:**

In the current study, multiple bioinformatics analyses were used to identify differentially expressed metabolic genes based on KRAS mutation status in PC. Then, we developed and validated a prognostic risk model based on the selected KRAS-associated metabolic genes. Besides, we explored the association between the risk model and the metabolic characteristics as well as gemcitabine-associated chemoresistance in PC.

**Results:**

6 KRAS-associated metabolic genes (i.e., CYP2S1, GPX3, FTCD, ENPP2, UGT1A10, and XDH) were selected and enrolled to establish a prognostic risk model. The prognostic model had a high C-index of 0.733 for overall survival (OS) in TCGA pancreatic cancer database. The area under the curve (AUC) values of 1- and 3-year survival were both greater than 0.70. Then, the risk model was validated in two GEO datasets and also presented a satisfactory discrimination and calibration performance. Further, we found that the expression of some KRAS-driven glycolysis-associated genes (PKM, GLUT1, HK2, and LDHA) and gemcitabine-associated chemoresistance genes (i.e., CDA and RMM2) was significantly upregulated in high-risk PC patients evaluated by the risk model.

**Conclusions:**

We constructed a risk model based on 6 KRAS-associated metabolic genes, which predicted patients' survival with high accuracy and reflected tumor metabolic characteristics and gemcitabine-associated chemoresistance in PC.

## 1. Introduction

Pancreatic cancer (PC) is one of the most malignant cancers, which leads to 4.5% of all cancer-related deaths globally [1]. The vital causes for the poor prognosis are the highly aggressive phenotype and early cancer recurrence and metastasis following surgical treatment [2]. Despite some advances in the management of PC in recent years, very few major breakthroughs for effective biomarkers nor treatment strategies have emerged.

Almost all PC patients carry at least one of the four frequently mutated driver genes, which include oncogene KRAS and the tumor suppressors TP53, SMAD4, and CDKN2A [3]. KRAS, the most frequently mutated oncogene in cancer especially in PC, was reported to rewire metabolism to support tumor growth [4]. Several studies showed that KRAS promoted metabolic reprogramming through the enhancement of glucose metabolism, differential channeling of glucose intermediates, reprogramming glutamine metabolism, and increasing autophagy and macropinocytosis [5, 6]. The metabolic reprogramming and fibrotic stroma of PC had been considered as a barrier for cytotoxic drug delivery to cancer cells, therefore contributing to chemoresistance and treatment failure [7, 8]. Although some KRAS-associated genes were found to regulate cancer cell metabolism, the mechanism still remained to be clarified and there was no comprehensive analysis to explore KRAS-associated metabolic signature or risk model for PC.

In the current study, multiple bioinformatics analyses were used to identify differentially expressed metabolic genes based on KRAS mutation status in PC. Then, a prognostic model was constructed based on the selected KRAS-associated metabolic genes. Moreover, we explored the association between the risk model and the metabolic characteristics and gemcitabine-associated chemoresistance in PC.

## 2. Materials and Methods

### 2.1. Datasets and Data Acquisition

**Figure 1:**
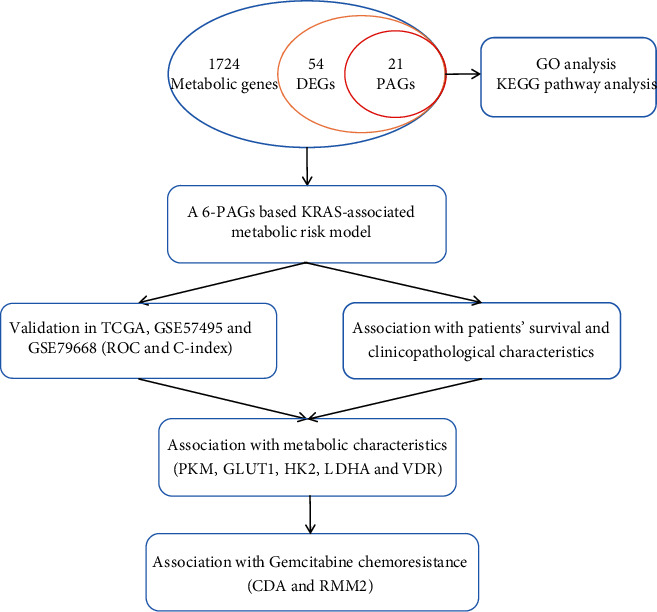
The flow chart summarizing the process of this study.

The gene expression data were recorded based on fragments per kilobase per million (FPKM), and clinical information for 178 PC samples was obtained from The Cancer Genome Atlas (TCGA, https://portal.gdc.cancer.gov/repository) database up to September 2020. The KRAS mutation data of TCGA PC cohort were downloaded from cBioPortal (http://www.cbioportal.org/) [9]. Among 178 PC samples, 167 PC samples with RNA-sequencing data and KRAS mutation information were subjected to subsequent analysis. The GSE57495 dataset based on GPL9115 (including 63 PC samples) and the GSE79668 dataset based on GPL11154 (including 51 PC samples) were downloaded from the Gene Expression Omnibus (GEO, https://www.ncbi.nlm.nih.gov/geo/) database for validation. Besides, two other GEO datasets, the GSE140077 dataset based on GPL20795 (including 1 PC cell line and 6 samples) and the GSE106336 dataset based on GPL18573 (including 1 PC cell line and 6 samples), were used for further study in gemcitabine resistance of PC. A total of 1724 metabolic genes were obtained from the metabolic pathway-related gene lists of “c2.cp.kegg.v7.1.symbols.gmt” in gene set enrichment analysis (GSEA, https://www.gsea-msigdb.org/gsea/index.jsp) [10]. Datasets mentioned above for PC were publicly obtainable, and ethics approval was not needed. One flow chart is displayed in Figure 1 to summarize the process of this study.

### 2.2. Differential Expression Analysis

We compared 58 PC samples without KRAS mutations (KRAS WT) and 109 PC samples with KRAS mutations (KRAS MUT) in TCGA PC cohort to identify differentially expressed metabolic genes (DEGs) using the R package limma. The thresholds were ∣log2 − fold change (FC) | >1.5 and FDR < 0.05.

### 2.3. Establishment and Validation of the KRAS-Associated Metabolic Risk Score System

With the help of R packages “survival,” “survminer,” and “parallel,” the univariate Cox proportional regression was performed to evaluate the association between expression of DEGs and patients' overall survival (OS) in TCGA cohort. DEGs with *p* value <0.01 were considered as statistically significant prognosis-associated DEGs (PAGs). Then, a multivariate Cox proportional regression model was established through cycle computation to determine the model with the highest discriminated ability for OS of PC. The KRAS-associated metabolic risk model was established based on a linear combination of the expression values of the PAGs, and the multivariate Cox regression coefficients were used as the weight. For the external validation cohort for the risk score system, the GSE57495 and GSE79668 datasets were analyzed. Patients in separate datasets were divided into a high-risk group and a low-risk group using the median cutoff of the risk score. To evaluate the performance of model, the R package “pROC” was used to evaluate the discrimination of the risk model in TCGA and GEO datasets through area under the curve (AUC) of the receiver operating characteristic (ROC) curve of 1- and 3-year survival. With the help of R package “rms,” Harrell's concordance index (C-index) of the risk score system was calculated by a bootstrap approach with 1000 resamples. The calibration curves were used to assess the consistency between model-predicted and observed survival. Then, the log-rank tests and Kaplan-Meier (KM) analyses were performed using the survival R package between the high-risk and low-risk groups to assess the predictive ability of the prognostic model. We calculated the risk scores in different groups based on clinicopathological parameters (such as American Joint Committee on Cancer (AJCC) stage, histologic grade, and diabetes status) in TCGA PC cohort by using Wilcoxon's test to evaluate whether the risk model reflects PC progression.

### 2.4. Functional Enrichment Analysis

Gene set enrichment analysis (GSEA, Version3.0, http://software.broadinstitude.org/gsea/) was performed to determine how the metabolic pathways and relevant metabolic pathway-related genes differed between PC samples of KRAS WT and KRAS MUT in TCGA cohort. An annotated gene set file (c2.cp.kegg.v7.1.symbols.gmt) was used as the reference gene set, and a gene set was considered as an enriched group when the nominal *p* value <0.05. DEGs and coexpressed genes of PAGs screened from the cBioPortal database were integrated to DAVID 6.7 (https://david-d.ncifcrf.gov/) separately to perform Gene Ontology (GO) analysis and Kyoto Encyclopedia of Genes and Genomes (KEGG) pathway analysis [11]. Results were visualized using R package “ggplot2.”

### 2.5. Association between the Risk Model and Metabolic Characteristics of PC

Several KRAS-driven metabolic targets which took part in metabolic reprogramming of PC were selected to explore relationships between the risk model and metabolic characteristics of PC [12]. The mRNA expressions levels of these promising KRAS-driven metabolic genes were compared in high-risk and low-risk groups by using Student's *t*-test.

### 2.6. Association between the Risk Model and Gemcitabine Chemoresistance of PC

In order to evaluate whether the risk model reflects gemcitabine metabolism-associated chemoresistance of PC, metabolic risk scores were calculated in 2 parental and GEM-resistant cell lines (CFPAC-1 and HPAFII) in GSE140077 and GSE106336 datasets. Then, several gemcitabine metabolism-associated chemoresistance genes of PC were selected to explore relationships between the risk model and gemcitabine chemoresistance of PC [13]. The mRNA expressions levels of these promising KRAS-driven metabolic genes were compared in high-risk and low-risk groups using Wilcoxon's test.

### 2.7. Statistical Analyses

All statistical analyses were performed using R software Version 4.0.1 (https://www.r-project.org/) and SPSS software Version 24.0 (SPSS, Inc., Chicago, IL, USA). X-tile (New Haven, CT, United States) was used to define the best cutoff values for outcome-based optimization. A *p* value <0.05 was considered statistically significant unless otherwise specified.

## 3. Results

### 3.1. Association between Metabolic Phenotype and KRAS Mutations in PC

**Figure 2:**
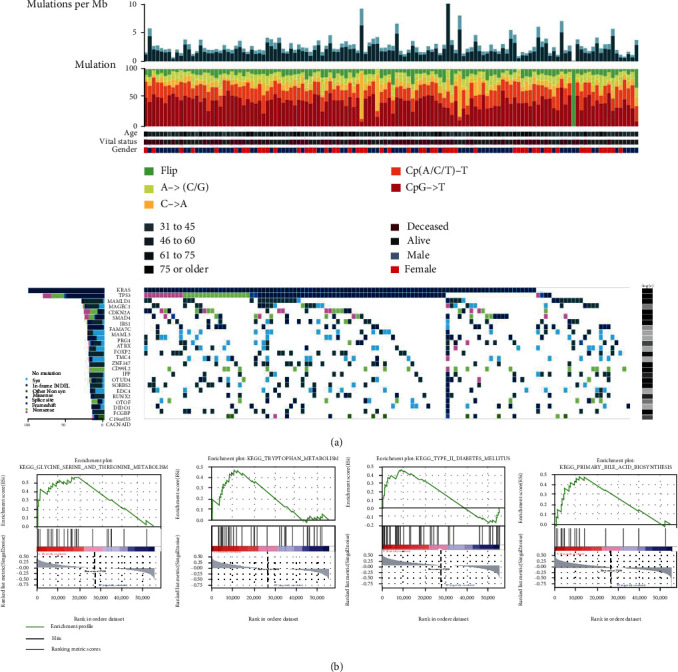
Gene set enrichment analysis (GSEA) of KRAS in TCGA pancreatic cancer (PC) cohort. (a) Genomic landscape and the mutational signatures of PC in TCGA cohort (FireBrowse platform). (b) Significant enrichment of the metabolic-related phenotype in KRAS WT PC patients compared with KRAS MUT PC patients.

It is shown in the FireBrowse Database (http://www.firebrowse.org/) that KRAS mutation was the most common type of mutation in PC, followed by mutation frequency of TP53, MAMLD1, MAGEC1, CDKN2A, and SMAD4 (Figure 2(a)). Next, we utilized mRNA expression data and corresponding clinical information of PC samples in TCGA and cBioPortal to investigate metabolic processes linked to KRAS mutation status. GSEA was conducted to determine the difference of metabolic pathways between 58 PC samples of KRAS WT and 109 PC samples of KRAS MUT. The results showed that 4 metabolic biological processes were significantly enriched in the KRAS MUT group, which were glycine serine and threonine metabolism (NES = 1.63, size = 31, and *p* value <0.05), tryptophan metabolism (NES = 1.48, size = 40, and *p* value <0.05), type II diabetes mellitus (NES = 1.41, size = 47, and *p* value <0.05), and primary bile acid biosynthesis (NES = 1.67, size = 16, and *p* value <0.05) (Figure 2(b)).

### 3.2. Identification and Enrichment Analysis of Differentially Expressed Metabolic Genes Based on KRAS Mutation Status

**Figure 3:**
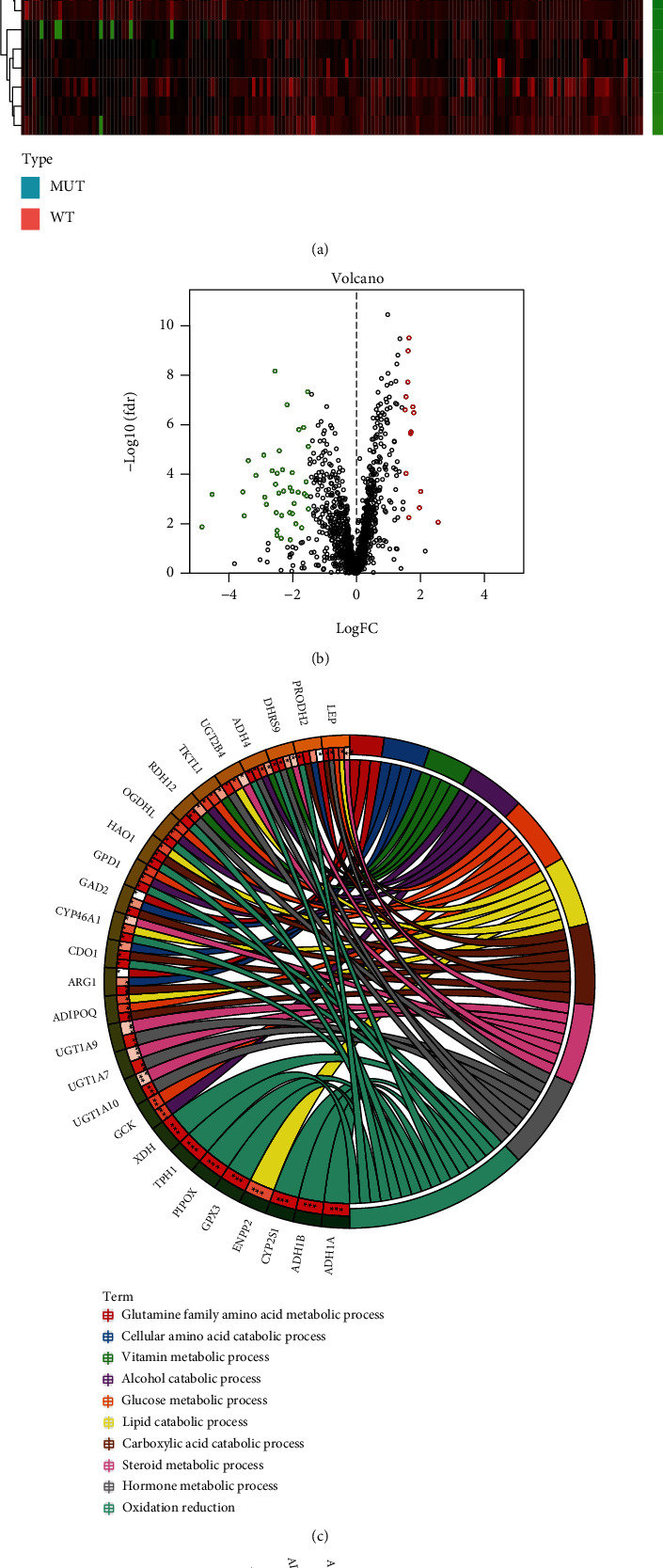
Identification and enrichment analysis of differentially expressed metabolic genes (DEGs) based on KRAS mutation status. (a) Heatmap and (b) volcano plot of 54 DEGs. (c) Gene Ontology (GO) enrichment analysis of DEGs. (d) Kyoto Encyclopedia of Genes and Genomes (KEGG) pathway analysis of DEGs.

We performed differential expression analyses between 58 PC samples of KRAS WT and 109 PC samples of KRAS MUT in TCGA PC cohort. Among 1724 metabolic genes, 54 genes were significant differentially expressed and considered as DEGs (∣log2 − fold change (FC) | >1.5 and FDR < 0.05) (Figures 3(a) and 3(b)). Then, these 54 DEGs were integrated to DAVID 6.7 to perform GO analysis and KEGG pathway analysis. GO analysis showed that the top 10 highly enriched functions in the metabolic process were “oxidation reduction, carboxylic acid catabolic process, hormone metabolic process, alcohol catabolic process, glucose metabolic process, lipid catabolic process, cellular amino acid catabolic process, vitamin metabolic process, steroid metabolic process, and glutamine family amino acid metabolic process” (Figure 3(c)). In KEGG analysis (Figure 3(d)), DEGs were found to be mainly enriched in “adipocytokine signaling pathway, retinol metabolism, metabolism of xenobiotics by cytochrome P450, type II diabetes mellitus, drug metabolism, endocytosis, and fatty acid metabolism.”

### 3.3. Construction and Validation of a KRAS-Associated Metabolic Risk Model

**Figure 4:**
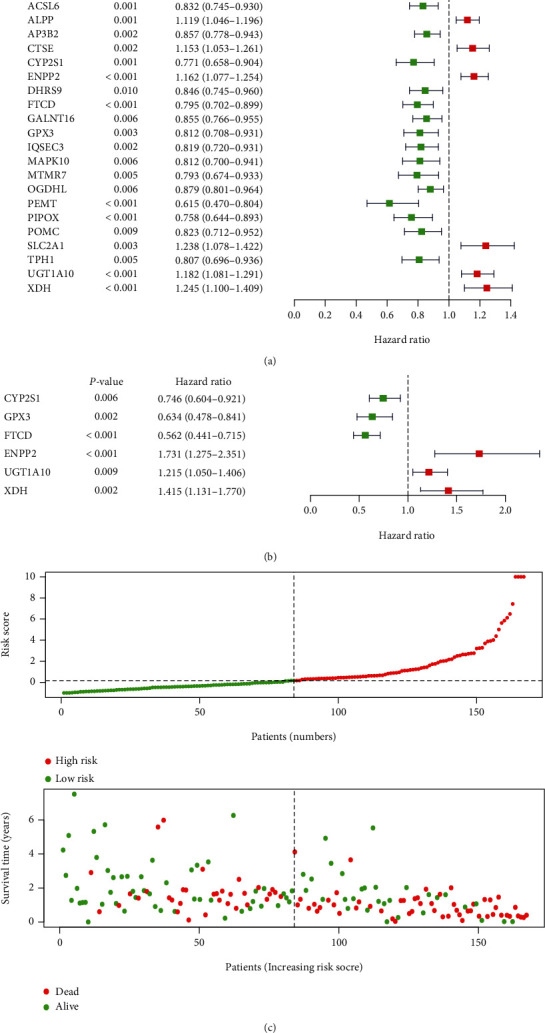
Construction of the KRAS-associated metabolic risk model for pancreatic cancer (PC). (a) Univariable and (b) multivariable Cox regression analyses to select prognosis-associated DEGs (PAGs). (c) Distribution of risk score and patient survival time and status of PC.

We performed univariate Cox proportional regression and found that 21 out of 54 DEGs were significantly related to OS and considered as prognosis-associated DEGs (PAGs) (Figure 4(a)). Further, with the help of the R package “survival,” a multivariate Cox regression analysis for these 21 PAGs was performed and it revealed that 6 of them were independently associated with OS (Figure 4(b)). Therefore, a 6-PAG-based KRAS-associated metabolic risk model was developed by weighting the normalized expression of these 6 PAGs multiplied by corresponding coefficients derived from the multivariate Cox regression analysis: risk score = (−0.29328∗normalized expression of CYP2S1) + (−0.45614∗normalized expression of GPX3) + (−0.57665∗normalized expression of FTCD) + (0.54899∗normalized expression of ENPP2) + (0.19472∗normalized expression of UGT1A10) + (0.34727∗normalized expression of XDH) (Table 1). Patients were divided into high-risk and low-risk groups by using the median cutoff value of the risk score (Figure 3(c)). The clinicopathological characteristics of patients in different risk groups were shown in Table 2.

**Figure 5:**
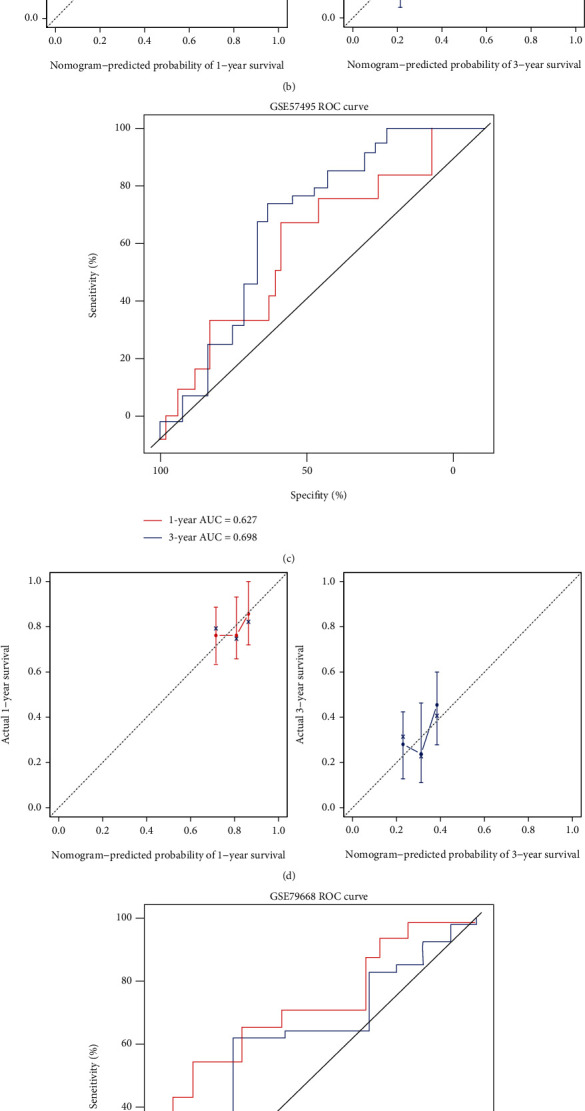
Prognostic abilities of the risk model. (a) Receiver operating characteristic (ROC) of the risk model for overall survival (OS) in TCGA cohort. Area under the curve (AUC) at the 1- and 3-year survival times was 0.773 and 0.704. (b) Calibration curves of the risk model for 1- and 3-year survival in TCGA cohort. (c) ROC of the risk model for OS in the GSE57495 dataset. AUC at the 1- and 3-year survival times was 0.627 and 0.698. (d) Calibration curves of the risk model for 1- and 3-year survival in the GSE79668 dataset. (e) ROC of the risk model for OS in the GSE57495 dataset. AUC at the 1- and 3-year survival times was 0.727 and 0.617. (f) Calibration curves of the risk model for 1- and 3-year survival in the GSE79668 dataset.

The predictive accuracy of the risk model was further assessed through the ROC and C-index analysis. The results showed the AUC of the risk model for OS in TCGA cohort was 0.773 at 1 year and 0.704 at 3 years (Figure 5(a)). Besides, C-index of the risk model in TCGA cohort was 0.733 (95% CI, 0.675-0.761). The calibration curves of the risk model matched well, which indicated that it could accurately predict the 1- and 3-year OS in TCGA cohort (Figure 5(b)). GSE57495 and GSE79668 datasets were used for the external validation for the risk model. The AUC for 1- and 3-year OS in GSE57495 datasets was 0.627 and 0.698 (Figure 5(a)), while the AUC for 1- and 3-year OS is 0.727 and 0.617 in GSE79668 datasets (Figure 5(e)). The C-index of the risk model in GSE57495 and GSE79668 datasets was 0.683 (95% CI, 0.655-0.723) and 0.625 (95% CI, 0.598-0.675). And the corresponding 1-year and 3-year calibration curves are, respectively, shown in Figures 5(d) and 5(f).

### 3.4. Association between the Risk Model and Patients' Survival and Clinicopathological Characteristics in PC

**Figure 6:**
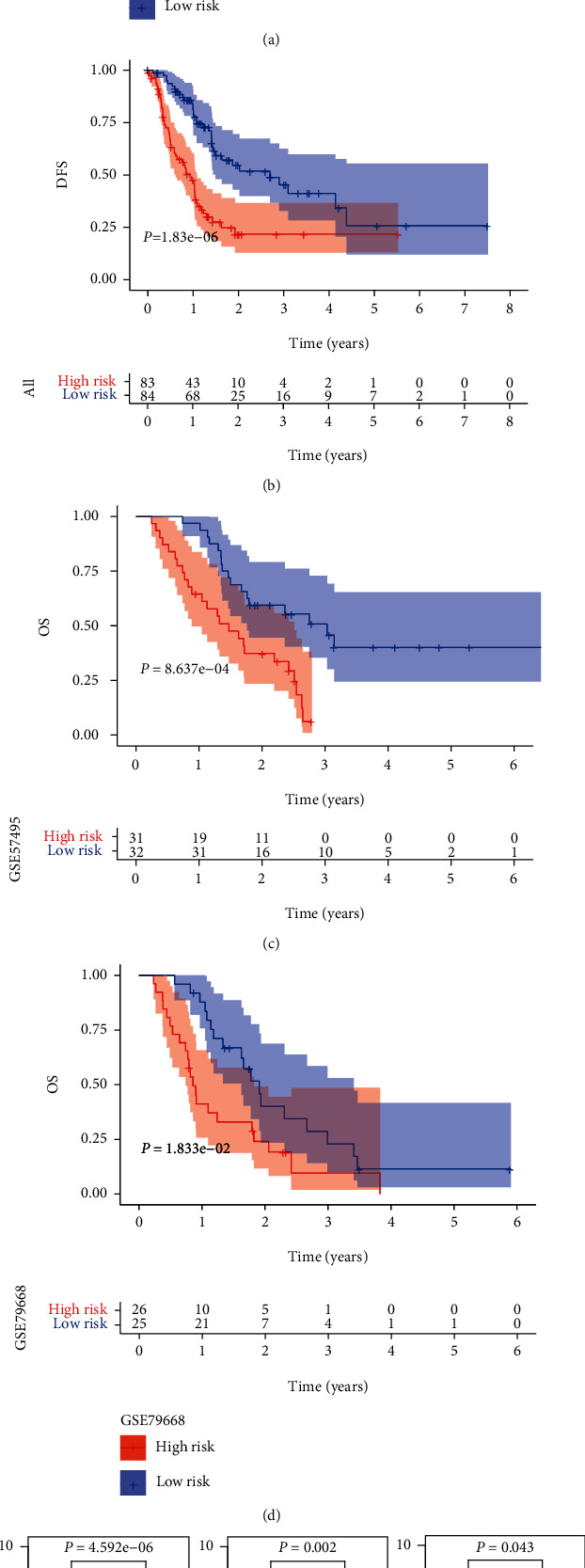
Association between the risk model and patients' survival and clinicopathological characteristics in pancreatic cancer (PC). (a, b) Kaplan-Meier (KM) analysis of TCGA pancreatic cancer patients was stratified by median risk. (a) Overall survival (OS) was significantly higher in the low-risk group than in the high-risk group. (b) Disease-free survival (DFS) was significantly higher in the low-risk group than in the high-risk group. (c) KM analysis of OS in the GSE57495 dataset. (d) KM analysis of OS in the GSE79668 dataset. (e) Patients with advanced stage (*p* value = 4.592*e*-06), higher histologic grade (*p* value = 0.002), or diabetes history (*p* value = 0.043) had higher risk scores.

To further evaluate the prognostic power of the risk model, Kaplan-Meier analyses were performed and we found that all patients in the high-risk group had a shorter OS (*p* value = 4.234*e*-07) and disease-free survival (DFS) (*p* value = 1.83*e*-06) than those in the low-risk group in TCGA cohort (Figures 6(a) and 6(b)). Besides, similar results for Kaplan-Meier analyses of OS were observed in the GSE57495 dataset (*p* value = 8.637*e*-04) and GSE79668 dataset (*p* value = 1.833*e*-02) (Figures 6(c) and 6(d)). Further, we calculated the risk scores in different groups based on clinicopathological characteristics in TCGA PC cohort, and the results showed that patients who had advanced stage, higher histologic grade, or diabetes history had higher risk scores (*p* value <0.05) (Figure 6(e)). The data in Figures 4 and 5 suggested the KRAS-associated metabolic risk model had effective value in predicting patients' survival and was associated with advanced tumor characteristics.

### 3.5. Association between the Risk Model and Metabolic Characteristics of PC

We next explored the activities of 6 PAGs (i.e., CYP2S1, GPX3, FTCD, ENPP2, UGT1A10, and XDH) by analyzing its potential metabolic pathways in PC. The coexpression analyses for 6 PAGs were performed by using the cBioPortal dataset (Spearman′s correlated coefficient > 0.6 or <−0.6, *p* value <0.05). We found 131 coexpression genes for CYP2S1, 163 coexpression genes for ENPP2, 387 coexpression genes for FTCD, 521 coexpression genes for GPX3, 189 coexpression genes for UGT1A10, and 213 coexpression genes for XDH, all of which were enrolled into DAVID 6.7 and subjected to functional and pathway enrichment analyses.

**Figure 7:**
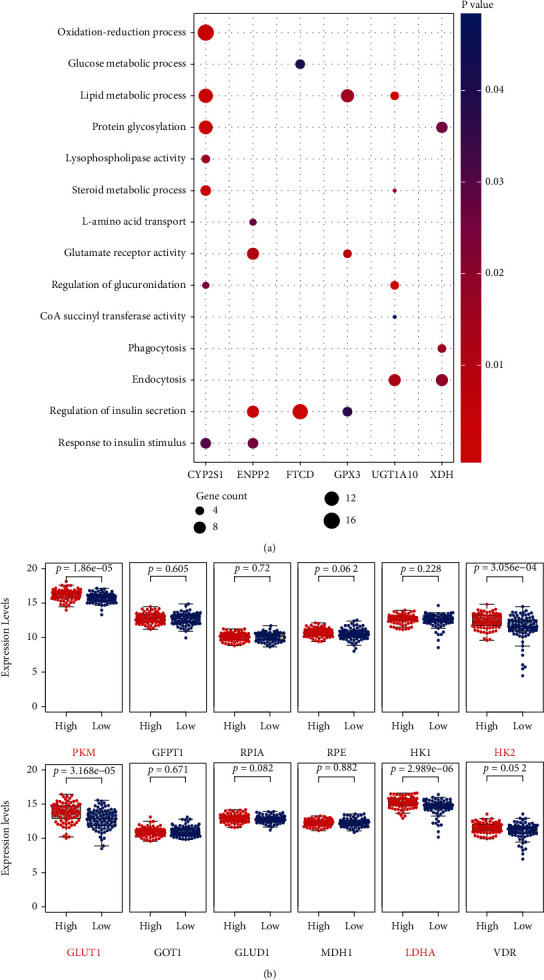
Association between the risk model and metabolic characteristics of pancreatic cancer (PC). (a) Enrichment analysis of 6 PAGs showed the potential metabolic pathways involved. (b) The expression levels of several KRAS-driven metabolic genes between high-risk and low-risk groups in TCGA cohort. Higher expressions of PKM (*p* = 1.86*e* − 05), GLUT1 (*p* = 3.168*e* − 05), HK2 (*p* = 3.056*e* − 04), LDHA (*p* = 2.989*e* − 06), and VDR (*p* = 0.012) were found in the high-risk group.

The potential metabolic pathways involved are shown in Figure 7(a). CYP2S1 and its neighboring genes were mainly enriched in “oxidation-reduction process, lipid metabolic process, protein glycosylation, lysophospholipase activity, steroid metabolic process, regulation of glucuronidation, and response to insulin stimulus.” GPX3 may act a vital role in “lipid metabolic process, glutamate receptor activity, and regulation of insulin secretion.” FTCD may play an important role in “glucose metabolic process and regulation of insulin secretion.” ENPP2 may be involved in “L-amino acid transport, glutamate receptor activity, regulation of insulin secretion, and response to insulin stimulus.” UGT1A10 was mainly associated with “lipid metabolic process, steroid metabolic process, regulation of glucuronidation, CoA succinyl transferase activity, and endocytosis,” and XDH was involved in “protein glycosylation, phagocytosis, and endocytosis.”

To further explore the potential metabolic targets influenced by our risk model, we compared the expression levels of several KRAS-driven metabolic genes between high-risk and low-risk groups in TCGA cohort. We found higher mRNA expressions of PKM (*p* = 1.86*e* − 05), GLUT1 (*p* = 3.168*e* − 05), HK2 (*p* = 3.056*e* − 04), LDHA (*p* = 2.989*e* − 06), and VDR (*p* = 0.012) in the high-risk group (Figure 7(b)).

### 3.6. Association between the Risk Model and Gemcitabine Chemoresistance in PC

**Figure 8:**
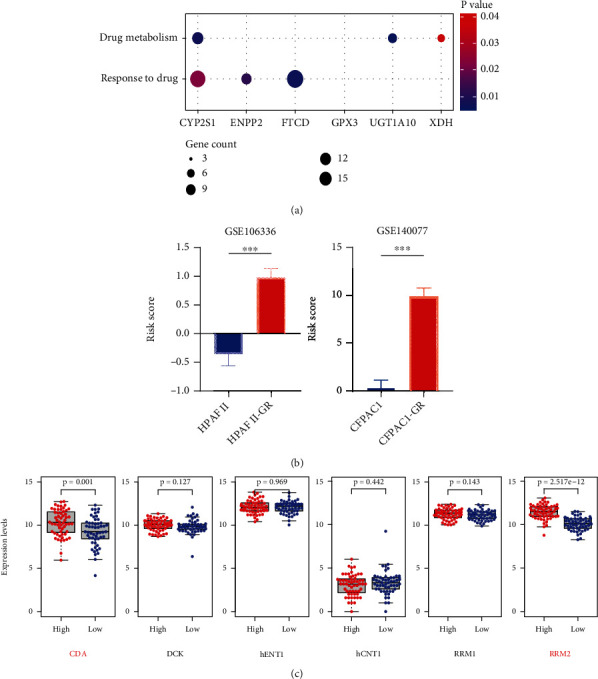
Association between the risk model and gemcitabine chemoresistance in pancreatic cancer (PC). (a) Enrichment analysis showed 5 of 6 prognosis-associated differentially expressed metabolic genes (PAGs) were involved in “drug metabolism or response to drug.” (b) Gemcitabine-resistant pancreatic cancer cell lines (CFPAC-1 and HPAFII) had higher risk scores than the parental group (*p* value <0.001). (c) The expression levels of several gemcitabine metabolism-associated chemoresistance genes between high-risk and low-risk groups in TCGA cohort. Higher expressions of CDA (*p* value = 0.001) and RMM2 (*p* value = 2.517*e*-12) were found in the high-risk group.

We found that 5 of 6 PAGs were enriched in “drug metabolism or response to drug” in functional and pathway enrichment analyses (Figure 8(a)). In order to explore their relationship with gemcitabine-associated chemoresistance in PC, we calculated metabolic risk scores in 2 parental gemcitabine-resistant cell lines (CFPAC-1 and HPAFII) in GSE140077 and GSE106336 datasets (Figure 8(b)). The results revealed that the gemcitabine-resistant group had significantly higher metabolic risk scores than the parental group (*p* value<0.001).

To investigate how the risk model leads to gemcitabine resistance, we compared the expression of previous reported genes regulating gemcitabine drug metabolism and efficacy (i.e., CDA, DCK, hENT1, hCNT1, RMM1, and RMM2) between high-risk and low-risk groups in TCGA cohort. The results showed that the mRNA levels of CDA (*p* value = 0.001) and RMM2 (*p* value = 2.517*e*-12) were significantly upregulated in the high-risk group (Figure 8(c)).

## 4. Discussion

As the most frequently mutated oncogene in PC, KRAS and its downstream pathways affect several cellular processes including cell proliferation, migration, metabolism, and autophagy in PC [14]. Small molecule drug (Sotorasib) that specifically and irreversibly inhibits KRAS had passed phase 1 clinical trial, showing a promising therapeutic prospect [15]. Studies have revealed oncogenic KRAS rewired metabolism to favor a more anabolic state and therefore promoted tumorigenesis and progression of PC. However, the molecular mechanisms were still not clear, and it is of great significance to conduct a profound study on KRAS-associated metabolic genes in PC.

Recent studies have offered ample insight into cancer metabolic landscapes to define PC. Gao et al. identified prognostic signatures and novel PC subtypes based on cancer metabolism using weighted gene coexpression network analysis (WGCNA) [16]. They also found higher simple nucleotide variant (SNV) frequencies of KRAS in the high-risk group. In the current study, we found out 6 differentially expressed metabolic genes based on KRAS mutation status, including CYP2S1, GPX3, FTCD, ENPP2, UGT1A10, and XDH. Then, we developed a significant KRAS-associated metabolic risk model for prognostic prediction in PC. Further, the risk model was validated in two external cohorts (GSE57495 and GSE79668 datasets), which showed a stably high prognostic value for PC. These results indicated our model was accurate in predicting patients' survival, which may be helpful in designing personalized therapy targeting metabolism reprogramming. In the risk model, high expression of CYP2S1, GPX3, and FTCD was correlated with better prognoses, while high expressions of ENPP2, UGT1A10, and XDH were correlated with poor prognoses in PC. As a member of cytochrome P450 (CYPs), CYP2S1 was known to biotransform some exogenous and endogenous compounds including drugs, fatty acids, and cholesterols [17]. Study showed that expressions of 8 CYPs were changed in CYP2S1-depleted cells, and all of them were involved in lipid biotransformation. Indeed, CYP2S1 affected the metabolism of arachidonic acid (AA) and linoleic acid (LA) to modulate BEAS-2B cell growth [18]. In consistent with their results, our data also found CYP2S1 was enriched in lipid metabolic process. It will be interesting to investigate the detailed mechanism by which CYP2S1 regulates lipid metabolism in PC in future study. As a member of glutathione peroxidase family (GPX), GPX3 reduces glutathione to catalyze the reduction of hydrogen peroxide, hydroperoxides, and lipid hydroperoxides [19]. Low GPX activity was found associated with enhanced lipid peroxidation in metastatic cancers, indicating that loss of GPX3 may promote systemic oxidative stress [20]. Studies had reported that low expression of GPX3 was associated with poor prognosis and chemoresistance in several tumor types [19]. Our results also proved that it is associated with a good outcome and may involve in “lipid metabolic process and glutamate receptor activity” in PC. ENPP2, which encodes an ecto-lysophospholipase D called autotaxin (ATX), was found significantly increased in several types of cancer including PC [21–23]. Overwhelming evidences revealed that the ATX/lysophosphatidate (LPA) signaling axis served key roles in energy metabolism regulation and obesity control, dysregulation of which could cause inflammation and tumorigenesis [24, 25]. Studies have indicated that ATX/LPA axis promoted DNA synthesis, proliferation, and invasion in pancreatic cancer cells via ERK1/2 and Rho pathways [26, 27]. Overexpression of ATX was also proven to lead elevated tumorigenesis and invasiveness compared with control groups in RAS-mutated NIH3T3 murine fibroblasts [28]. Further studies are needed to explore the relationship and underlying regulation mechanism of KRAS mutation and ENPP2/ATX in PC tumorigenesis and metabolism reprogramming.

UGT1A10 was a extrahepatic phase II metabolizing enzyme that expressed highly in numerous target areas for tobacco-induced cancers, including the upper aerodigestive tract [29]. UGT1A10 was elevated in a CPT-11/SN-38-resistant cell line and responsible for SN-38 glucuronidation, which was one of the mechanisms associated with irinotecan hydrochloride/7-ethyl-10-hydroaxycamptothecin (CPT-11/SN-38) resistance in lung cancer [30]. FTCD was found significantly downregulated in hepatocellular carcinoma (HCC) and served as a diagnostic biomarker for HCC [31]. Recently, FTCD was found to be associated with the sensitivity of chemotherapeutic drug methotrexate by using a CRISPR-Cas9-based screen [32]. XDH, a rate-limiting enzyme to catalyze the final steps of purine metabolism, was found significantly decreased and served as a useful predictor of poor prognoses in several cancer types [33]. Uric acid, which was transformed by XDH, was found to modulate tumor cell sensitivity to the antimetabolite 5-FU, one of the most commonly used anticancer drugs in the clinic [34]. Our study consistently suggested that UGT1A10, FTCD, and XDH were involved in “drug metabolism or response to drug” in PC, but their specific roles in metabolic reprogramming and chemoresistance in PC still remain to be studied.

Gemcitabine-based chemotherapy is a major treatment for PC patients with or without surgical resection. It has been reported gemcitabine drug metabolism was affected by some genes or metabolites, which included drug transporters (i.e., hEN1 and hCNT1), activating and inactivating enzymes (i.e., CDA and DCK), and competitive substrates to active metabolites (i.e., RRM1 and RRM2) [13]. Cytidine deaminase (CDA) induced the deamination of dFdC to dFdU, leading to inactivation of gemcitabine [35]. It had been confirmed that CDA expression was correlated with OS in PC, and several in vitro studies revealed that overexpression of CDA led to gemcitabine resistance, while loss of CDA recovered gemcitabine sensitivity [36, 37]. RRM2 was involved in the activity of Ribonucleotide Reductase (RR), which was a rate-limiting enzyme of DNA synthesis. It has been demonstrated that upregulation of RRM2 led to gemcitabine chemoresistance in PC cells and human PC xenografts in mice [38]. Besides, expression level of RRM2 was correlated inversely with OS in gemcitabine-treated PC patients in clinical study [39]. In the current study, CDA and RRM2 were upregulated in the high-risk group evaluated by the risk model. Taken these together, the risk model may reflect the possibility of gemcitabine resistance, which would help oncologist choose appropriate chemotherapy regimen for PC.

There are a few limitations of our study. First, our study is mainly based on bioinformatics analysis, and more experimental studies are needed to investigate how KRAS may regulate cancer cell metabolism in PC. Second, the established model needs to be further validated in prospective clinical studies.

In conclusion, we constructed and validated a prognostic model based on the KRAS-associated metabolic genes in PC. The prognostic model reflects tumor metabolic characteristics and gemcitabine-associated chemoresistance, which may have important value in aiding personalized therapy.

## Figures and Tables

**Table 1:** 6 KRAS-associated metabolic genes to establish the risk model.

Genes	coef	HR	HR.95L	HR.95H	*p* value
CYP2S1	-0.29328	0.74582	0.60422	0.92060	0.00633
GPX3	-0.45614	0.63372	0.47754	0.84098	0.00158
FTCD	-0.57665	0.56178	0.44136	0.71504	2.80*E*-06
ENPP2	0.54899	1.73150	1.2754	2.3506	0.000432
UGT1A10	0.19472	1.21498	1.0496	1.4064	0.00911
XDH	0.34727	1.41520	1.1314	1.7702	0.00236

**Table 2:** The clinicopathological characteristics of patients in different risk groups.

Clinicopathological variables	Patients (*n* = 164)	Risk group		*p* value
		High (82)	Low (82)	
Alcohol history				0.15
Present	96	53	43	
Absent	68	29	39	
Diabetes				0.026
Present	48	31	17	
Absent	116	51	65	
Tumor size (cm)				0.43
<4	86	40	46	
≧4	78	42	36	
Lymphnode metastasis				0.38
Present	118	62	56	
Absent	46	20	26	
Distant metastasis				1
Present	92	46	46	
Absent	72	36	36	
TNM stage				0.00046
Advanced (IIA, III, & IV)	119	70	49	
Early (I & IIA)	45	12	33	
Differentiation				0.00055
Poor	47	34	13	
Well	117	48	69	

## Data Availability

The data that support the findings of this study are available from TCGA (https://portal.gdc.cancer.gov/repository), GEO (GEO, https://www.ncbi.nlm.nih.gov/geo/), and GSEA databases (https://www.gsea-msigdb.org/gsea/index.jsp).

## Abbreviations

PC: Pancreatic cancer
OS: Overall survival
AUC: Area under the curve
FPKM: Fragments per kilobase per million
TCGA: The Cancer Genome Atlas
GEO: Gene Expression Omnibus
GSEA: Gene set enrichment analysis
DEGs: Differentially expressed genes
PAGs: Prognosis-associated DEGs
ROC: Receiver operating characteristic
AJCC: American Joint Committee on Cancer
GO: Gene Ontology
KEGG: Kyoto Encyclopedia of Genes and Genomes.

## References

[1] Kamisawa T., Wood L. D., Itoi T., Takaori K. (2016). Pancreatic cancer. *Lancet*.

[2] Singhi A. D., Koay E. J., Chari S. T., Maitra A. (2019). Early detection of pancreatic cancer: opportunities and challenges. *Gastroenterology*.

[3] Cicenas J., Kvederaviciute K., Meskinyte I., Meskinyte-Kausiliene E., Skeberdyte A., Cicenas J. (2017). KRAS, TP53, CDKN2A, SMAD4, BRCA1, and BRCA2 mutations in pancreatic cancer. *Cancers (Basel)*.

[4] Tape C. J., Ling S., Dimitriadi M. (2016). Oncogenic KRAS regulates tumor cell signaling via stromal reciprocation. *Cell*.

[5] White E. (2013). Exploiting the bad eating habits of Ras-driven cancers. *Genes & Development*.

[6] Kimmelman A. C. (2015). Metabolic dependencies in RAS-driven cancers. *Clinical Cancer Research*.

[7] Dimou A., Syrigos K. N., Saif M. W. (2012). Overcoming the stromal barrier: technologies to optimize drug delivery in pancreatic cancer. *THER ADV MED ONCOL*.

[8] Lin J., Xia L., Liang J. (2019). The roles of glucose metabolic reprogramming in chemo- and radio-resistance. *Journal of Experimental & Clinical Cancer Research*.

[9] Gao J., Aksoy B. A., Dogrusoz U. (2013). Integrative analysis of complex cancer genomics and clinical profiles using the cBioPortal. *Science Signaling*.

[10] Subramanian A., Tamayo P., Mootha V. K. (2005). Gene set enrichment analysis: a knowledge-based approach for interpreting genome-wide expression profiles. *Proceedings of the National Academy of Sciences of the United States of America*.

[11] Huang D. W., Sherman B. T., Lempicki R. A. (2009). Systematic and integrative analysis of large gene lists using DAVID bioinformatics resources. *Nature Protocols*.

[12] Kawada K., Toda K., Sakai Y. (2017). Targeting metabolic reprogramming in KRAS-driven cancers. *International Journal of Clinical Oncology*.

[13] Amrutkar M., Gladhaug I. P. (2017). Pancreatic cancer chemoresistance to gemcitabine. *Cancers (Basel)*.

[14] Lanfredini S., Thapa A., O'Neill E. (2019). RAS in pancreatic cancer. *Biochemical Society Transactions*.

[15] Iacobuzio-Donahue C. A., Maitra A., Olsen M. (2003). Exploration of global gene expression patterns in pancreatic adenocarcinoma using cDNA microarrays. *The American Journal of Pathology*.

[16] Gao Y., Zhang E., Fei X., Kong L., Liu P., Tan X. (2021). Identification of novel metabolism-associated subtypes for pancreatic cancer to establish an eighteen-gene risk prediction model. *Frontiers in Cell and Development Biology*.

[17] Yan P., Eng O. C., Yu C. J. (2018). A review on the expression and metabolic features of orphan human cytochrome P 450 2S1 (CYP2S1). *Current Drug Metabolism*.

[18] Madanayake T. W., Lindquist I. E., Devitt N. P., Mudge J., Rowland A. M. (2013). A transcriptomic approach to elucidate the physiological significance of human cytochrome P450 2S1 in bronchial epithelial cells. *BMC Genomics*.

[19] Chang C., Worley B. L., Phaëton R., Hempel N. (2020). Extracellular glutathione peroxidase GPx3 and its role in cancer. *Cancers (Basel)*.

[20] Guven M., Ozturk B., Sayal A., Ozeturk A., Ulutin T. (1999). Lipid peroxidation and antioxidant system in the blood of cancerous patients with metastasis. *Cancer Biochemistry Biophysics*.

[21] Lee S. C., Fujiwara Y., Liu J. (2015). Autotaxin and LPA1 and LPA5 receptors exert disparate functions in tumor cells versus the host tissue microenvironment in melanoma invasion and metastasis. *Molecular Cancer Research*.

[22] Wu J. M., Xu Y., Skill N. J. (2010). Autotaxin expression and its connection with the TNF-alpha-NF-*κ*B axis in human hepatocellular carcinoma. *MOL CANCER*.

[23] Kadekar S., Silins I., Korhonen A. (2012). Exocrine pancreatic carcinogenesis and autotaxin expression. *PLoS One*.

[24] Barbayianni E., Kaffe E., Aidinis V., Kokotos G. (2015). Autotaxin, a secreted lysophospholipase D, as a promising therapeutic target in chronic inflammation and cancer. *Progress in Lipid Research*.

[25] Benesch M. G., Ko Y. M., McMullen T. P., Brindley D. N. (2014). Autotaxin in the crosshairs: taking aim at cancer and other inflammatory conditions. *FEBS Letters*.

[26] Ryder N. M., Guha S., Hines O. J., Reber H. A., Rozengurt E. (2001). G protein-coupled receptor signaling in human ductal pancreatic cancer cells: neurotensin responsiveness and mitogenic stimulation. *Journal of Cellular Physiology*.

[27] Takiguchi S., Nishino Y., Inoue K. (2012). The bisphosphonate incadronate inhibits intraperitoneal dissemination in an in vivo pancreatic cancer model. *Oncology Reports*.

[28] Liu S., Umezu-Goto M., Murph M. (2009). Expression of autotaxin and lysophosphatidic acid receptors increases mammary tumorigenesis, invasion, and metastases. *Cancer Cell*.

[29] Balliet R. M., Chen G., Dellinger R. W., Lazarus P. (2010). UDP-glucuronosyltransferase 1A10: activity against the tobacco-specific nitrosamine, 4-(methylnitrosamino)-1-(3-pyridyl)-1-butanol, and a potential role for a novel UGT1A10 promoter deletion polymorphism in cancer susceptibility. *Drug Metabolism and Disposition*.

[30] Oguri T., Takahashi T., Miyazaki M. (2004). UGT1A10 is responsible for SN-38 glucuronidation and its expression in human lung cancers. *Anticancer Research*.

[31] Chen J., Chen Z., Huang Z., Yu H., Li Y., Huang W. (2019). Formiminotransferase cyclodeaminase suppresses hepatocellular carcinoma by modulating cell apoptosis, DNA damage, and phosphatidylinositol 3-kinases (PI3K)/Akt signaling pathway. *Medical Science Monitor*.

[32] Kanarek N., Keys H. R., Cantor J. R. (2018). Histidine catabolism is a major determinant of methotrexate sensitivity. *Nature*.

[33] Saidak Z., Louandre C., Dahmani S. (2018). A pan-cancer study of the transcriptional regulation of uricogenesis in human tumours: pathological and pharmacological correlates. *Bioscience Reports*.

[34] Cantor J. R., Abu-Remaileh M., Kanarek N. (2017). Physiologic medium rewires cellular metabolism and reveals uric acid as an endogenous inhibitor of UMP synthase. *Cell*.

[35] Shipley L. A., Brown T. J., Cornpropst J. D., Hamilton M., Daniels W. D., Culp H. W. (1992). Metabolism and disposition of gemcitabine, and oncolytic deoxycytidine analog, in mice, rats, and dogs. *Drug Metabolism and Disposition*.

[36] Weizman N., Krelin Y., Shabtay-Orbach A. (2014). Macrophages mediate gemcitabine resistance of pancreatic adenocarcinoma by upregulating cytidine deaminase. *Oncogene*.

[37] Maréchal R., Bachet J. B., Mackey J. R. (2012). Levels of gemcitabine transport and metabolism proteins predict survival times of patients treated with gemcitabine for pancreatic adenocarcinoma. *Gastroenterology*.

[38] Jonckheere N., Skrypek N., Merlin J. (2012). The mucin MUC4 and its membrane partner ErbB2 regulate biological properties of human CAPAN-2 pancreatic cancer cells via different signalling pathways. *PLoS One*.

[39] Nakai Y., Isayama H., Sasaki T. (2012). A multicentre randomised phase II trial of gemcitabine alone _vs_ gemcitabine and S-1 combination therapy in advanced pancreatic cancer: GEMSAP study. *British Journal of Cancer*.

